# Determination of the Water Potential Threshold at Which Rice Growth Is Impacted

**DOI:** 10.3390/plants7030048

**Published:** 2018-06-22

**Authors:** Caio Luiz dos Santos, André Froes de Borja Reis, Paulo Mazzafera, José Laércio Favarin

**Affiliations:** 1 Departament of Crop Science, College of Agriculture Luiz de Queiroz, University of São Paulo, CP 09, Piracicaba, SP 13418-900, Brazil; santos.cl@outlook.com (C.L.d.S.); pmazza07@gmail.com (P.M.); favarin.esalq@usp.br (J.L.F.); 2 Department of Plant Biology, Institute of Biology, University of Campinas, Campinas, SP 13083-862, Brazil

**Keywords:** *Oryza sativa*, irrigation, polyethylene glycol 6000, water potential, carbon assimilation

## Abstract

Rice feeds 50% of the world’s population. Flooding is the most common irrigation system used for growing rice, a practice responsible for a large amount of water loss. Climate changes may affect water availability in irrigated agriculture, and it will be necessary to develop more sustainable irrigation practices. The aim of this work was to determine, in controlled conditions, the threshold when water potential begins to decrease plant growth. Two independent greenhouse experiments were conducted during middle summer and fall, in order to validate the results for high and low evapotranspiration conditions. Rice plants were grown in hydroponics and the water potential was adjusted with polyethylene glycol 6000, varying from −0.04 MPa (control) to −0.19 MPa. Leaf water potential, water use efficiency, leaf area, and root and shoot biomass were evaluated. All assayed parameters decreased as the water potential was decreased. The water potential threshold which starts to negatively affect rice growth was between −0.046 and −0.056 MPa, which are values close to those observed in the field in previous research. The definition of a critical value may help to improve water management in rice cultivation and to maintain productivity.

## 1. Introduction

Rice (*Oryza sativa*) is one of the main crops worldwide, and is grown on approximately 160 million hectares. The largest producing region is Asia, responsible for 90% of the 741 million tons produced annually [1]. Brazil is the ninth largest producer of rice in the world, and produces around 1.7% of the world’s rice: 11.7 million tons on 2.2 million hectares. In Brazil, 60% of rice production is irrigated by flooding.

Particularly when the world is facing prospects of climate change and water scarcity, and given the expressivity of rice, it is important to discuss the rational use of water in agriculture. Moreover, the flood irrigation system has several losses of water, such as direct evaporation on the atmosphere–water interface, lateral flow, and deep percolation [2]. These losses significantly increase the amount of water to be used in irrigation. Rice evapotranspiration ranges from 4 to 5 mm day^−1^ in the rainy season, and up to 7 mm day^−1^ in the dry season [2]. Total water losses are around 10 mm day^−1^ [3]. Clearly, science needs to move forward when discussing the use of water in agriculture. More efficient and sustainable irrigation systems for growing rice must be found [4].

Aiming to achieve this goal, several types of research have proposed changes in rice irrigation systems. Among them, there are three main strategies: aerobic system, where the plants are rain-fed or sprinkler-watered [5,6,7,8,9]; keep the soil saturated, but with no superficial water [10,11,12]; and intermittent flooding [4,6,13,14,15,16]. However, each of these strategies that have been chosen can potentially decrease yield with less availability of water in critical stages [17] because rice is very sensitive to drought [18,19]. Alongside modern efficient irrigation methods, drought-tolerant rice varieties have been bred [20], aiming to reduce water use. Since drought tolerance is strongly linked to plant height, breaking down this linkage has resulted in dwarf varieties [21], and it is very feasible that the new varieties will have marked changes on physiological processes related with the drought tolerance. Rice is also known to have a poor development of the root system limiting water uptake from unsaturated soil [22].

The main studies proposing a critical water potential for rice obtained values between −0.02 and −0.25 MPa [23,24,25]. In the referred studies, these parameters were obtained in varieties different from those that are currently in use in many tropical regions. It is assumed that the evapotranspiration is unchanged when the moisture’s water potential is bigger than −0.1 MPa, and that the leaf expansion stops completely when it is between −0.05 and −0.25 MPa, which varies with the phenological stage.

Recently, Reis et al. [4] studied different irrigation systems for lowland rice in Brazil in two subsequent years. They verified that productivity was maintained in the first year (2016) when soil water potential was between −0.02 and −0.03 MPa, but decreased in the following year (2017) when it reached −0.05 to −0.06 MPa during the vegetative (V4–V5) and reproductive (R0–R4) developmental stages. Thus, considering this previous result, and the importance of water availability to rice cultivation and the challenges of food production worldwide, establishing the critical water potential for rice and how yield is affected may help further studies on non-flooded water cultivation systems. Thus, the aim of this work was to confirm, in controlled conditions, the threshold when water potential begins to decrease plant growth.

## 2. Material and Methods

Greenhouse experiments were established in Piracicaba, SP—Brazil (23°07′ S; 47°38′ W and 578 m altitude) during the summer (February) and fall (May) of 2017. The local climate is classified as Cfa—humid subtropical without dry season [26] with an average annual temperature of 26.7 °C. Rice plants of IRGA 424 lowland variety were nursing-germinated, and 28-day-old seedlings were transplanted into 3 litre pots (0.14 m diameter × 0.3 m height). On the top of each pot, a 15 mm thick layer of Styrofoam, with a hole in the center, was placed to provide sustentation to the seedlings and prevent the evaporation of the nutrient solution. The plants were grown in nutrient solution containing: N—210 mg·L^−1^; P—31 mg·L^−1^; K—234 mg·L^−1^; Ca—200 mg·L^−1^; Mg—48 mg·L^−1^; S—64 mg·L^−1^; B—0.5 mg·L^−1^; Mn—0.5 mg·L^−1^; Zn—0.05 mg·L^−1^; Cu—0.02 mg·L^−1^; Mo—0.01 mg·L^−1^; Fe—5 mg·L^−1^; Cl—0.7 mg·L^−1^. The water potential of this nutrient solution was −0.04 MPa, calculated by the Van’t Hoff equation [27]. Electrical conductivity and pH of the solution were monitored every 10 days with a portable Hanna pH and conductivity tester (model HI98129). The pH ranged between 6 and 6.5. The electrical conductivity did not exceed 2.3 mS cm^−1^.

Plants were exposed to two evapotranspiration conditions, each one in a cycle of 45 days. The first was from 1 February to 23 March, and will be referred to as high evapotranspiration (HET). The second was from 26 April to 9 June, and will be referred as low evapotranspiration (LET). The climate conditions among growing cycles are indicated in Table 1.

Polyethylene glycol (PEG) 6000 was used to change the water potential of the nutritive solution. PEG 6000 is a high molecular weight polymer, not absorbable by plants, highly soluble in water, non-toxic, and it has no saline effect [28,29]. The levels of PEG 6000 were calculated with the equation described by [30]: Ψw = −(1.18 × 10^−2^)*C* − (1.18 × 10^−4^)*C*^2^ + (2.67 × 10^−4^)*CT* + 8.39 × 10^−7^)*C*^2^*T*, where Ψw = water potential of the nutrient solution (bar), *C* = concentration of PEG6000 (g L^−1^), T = temperature (°C). Plants were subjected to six levels of water potential: T1: −0.4 Bar (−0.04 MPa); T2: −0.7 Bar (−0.07 Mpa); T3: −1.0 Bar (−0.10 MPa); T4: −1.3 Bar (−0.13 Mpa); T5: −1.6 Bar (−0.16Mpa); T6: −1.9 Bar (−0.19 Mpa) starting on the day the seedlings were transplanted to the hydropony. At 45 days after transfer (DAT) to nutrient solutions, control plants reached V4–R3 phenological stages [31]. The experiment was conducted in a randomized block design, with four replications. Each plant represented a plot, and the number of plots was 24 per each evapotranspiration condition.

Evapotranspiration was considered the volumetric variation of nutritive solution volume in a day interval. Nutrient solution was replenished to the maximum each time the volume dropped approximately 150 mL (on average, 3 days). The total evapotranspiration for the period was determined by the sum of the completed volumes.

Also, in the 45 DAT, the leaf water potential was determined in the youngest mature leaf at 13:00. On the HET, this parameter was measured using a Scholander pressure chamber (model 3115, Soil Moisture Equipment, Goleta, CA, USA). On LET, it was measured with a thermocouple psychrometer (model PSYPRO P3-765, Wescor, Logan, UT, USA). The leaf water potential of treatment 6 was not measured on HET, because the plants did not have enough leaves for the analysis.

At the end of the LET and HET experiments, the plants were evaluated for leaf area and aboveground and root biomass. The leaf area was measured in a leaf area integrator (model 3400, LiCor, Lincoln, NE, USA) immediately after being cut in order to avoid curling leaves. The biomass was obtained after 72 h drying at 60 °C. Leaf-specific weight (LSW) and water use efficiency (WUE) were obtained by dividing the aboveground biomass by the leaf area and volume of water consumed during the treatment period, respectively [32].

The data were tested for homogeneity of variance and error normality assumptions by PROC TRANSREG Boxcox statement (SAS 9.2, Cary, NC, USA). If necessary, the variable was transformed by convenient lambda. Nonlinear regressions were adjusted, and its significance tested using Sigmaplot software (v 10.0, San Jose, CA, USA). The difference between treatments was considered significant if the 95% confidence intervals did not overlap [33].

## 3. Results

### 3.1. Evapotranspiration and Leaf Area

**Figure 1:**
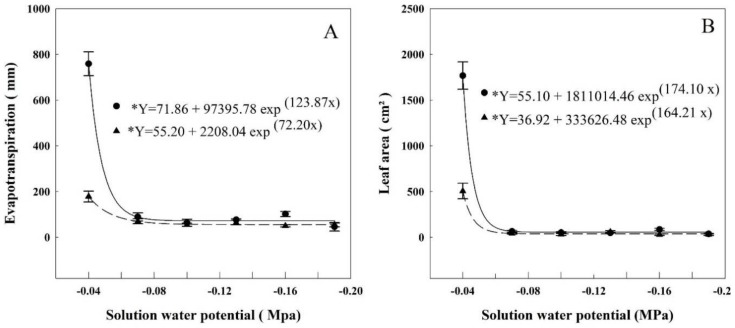
Evapotranspiration (**A**) and leaf area (**B**) of rice plants grown at different solutions with different water potentials. The error bars represent the standard error. * *p*-value < 0.01; • = HET; ▲ = LET.

At HET conditions, the total evapotranspiration (Figure 1) was 758 mm, on average, when the water potential of the solution was at the highest potential (−0.04 MPa). The water use decreased 88% when compared to the next level of water potential (−0.07 MPa) and from then on, it did not vary, averaging 76 mm. The exponential regression fitted to the data indicates that the evapotranspiration started to decrease when the water potential was lower than −0.048 MPa. At LET, the average evapotranspiration was 179 mm when the water potential of the solution was −0.04 MPa. There was also a sharp decrease in the next water potential, −0.07 MPa, and from then on, did not vary, averaging 58 mm evapotranspiration. The regression verifies that the interference of solution water potential started at −0.047 MPa.

Similar curves were obtained for leaf area in both HET and LET. The leaf area was 1770 cm^2^ at HET and 505.4 cm^2^ at LET, then sharply dropping in the next water potential −0.07 MPa. After that, they did not vary, averaging 57 cm^2^ in HET and 37.6 cm^2^ in LET. The exponential regression determines that the leaf area decrease began at −0.046 MPa and 0.053 MPa in HET and LET, respectively.

### 3.2. Plant Biomass

**Figure 2:**
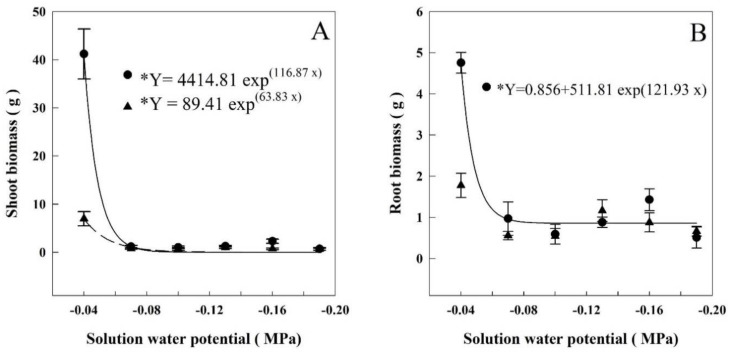
Shoot (**A**) and root (**B**) biomasses of rice plants grown at different solutions with different water potentials. The error bars represent the standard error. * *p*-value < 0.01; • = HET; ▲ = LET.

At HET and LET, the shoot biomass decreased on average 97% and 89% between −0.04 and −0.07 MPa, dropping from 41.2 to 1.3 g and 6.97 to 0.76 g, respectively (Figure 2A). The regressions adjusted for each period showed that the shoot biomass decreased when the solution water potentials were lower than −0.056 and −0.057 MPa, respectively.

The root biomass at HET period was decreased, on average, 81% between −0.04 and −0.07 MPa. It went from 4.75 g to 0.87 g, respectively. (Figure 2B). The regression adjusted for HET showed that the root biomass decreased when the solution water potential went lower than −0.063 MPa.

### 3.3. Leaf Water Potential

**Figure 3:**
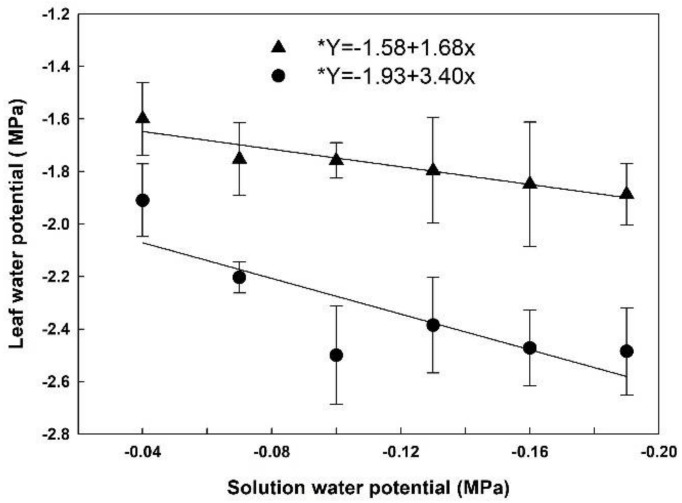
Leaf water potential at 13:00 in rice plants grown at different solutions with different water potentials. The error bars represent the standard error. * *p*-value < 0.05; • = HET; ▲ = LET.

Leaf water potential was lower in HET than in LET at 13:00, and in both cases, it decreased as the solution water potential was decreased (Figure 3). At 13:00, leaf water potential in HET plants averaged −2.40 MPa, and −1.80 MPa in LET plants. We also measured leaf water potential at 6:00, and although HET plants had lower values than LET, the data did not differ among the solution water potentials applied in both cases. This suggests that plants recovered from water stress during the night (data not shown).

### 3.4. Specific Leaf Weight

**Figure 4:**
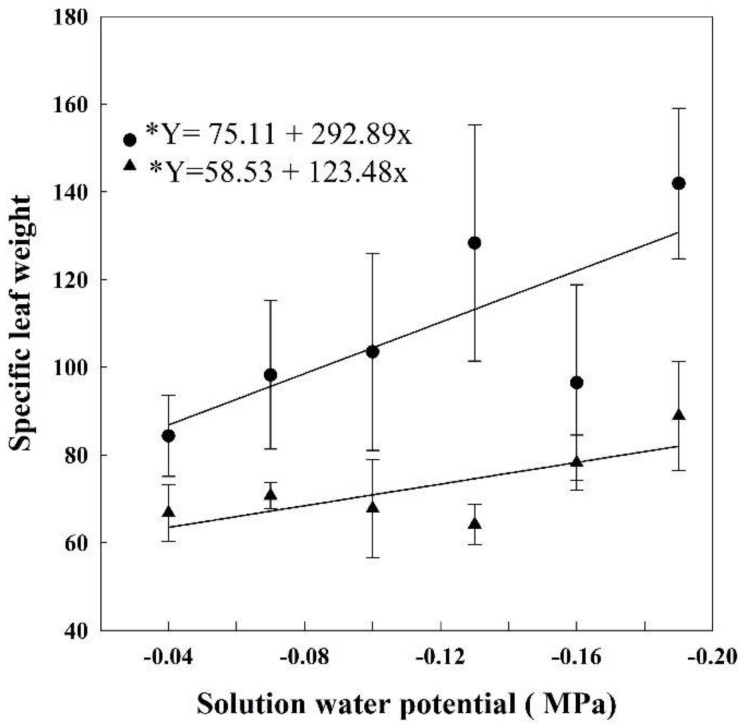
Specific leaf weight in rice plants grown at different solutions with different water potentials. The error bars represent the standard error. * *p*-value < 0.01; • = HET; ▲ = LET.

As the solution water potential decreased the specific leaf weight (SLW) of HET and LET plants increased (Figure 4). During HET, the SLW was 84.3 g m^−2^ in plants grown at −0.04 MPa, but at −0.19 MPa, it was 141.9 g m^2^, 67% bigger. At LET, the SLW of the plants grown at −0.04 MPa was 66.7 g m^−2^, reaching 88.9 g m^−2^ at −0.19 MPa, a 33% increase.

### 3.5. Water Use Efficiency

In both HET and LET, the WUE dropped drastically from −0.04 MPa to −0.07 MPa (Table 2). At HET, the average of the plants grown at −0.04 MPa was 3.47 mg biomass g^−1^ H_2_O and averaged to 1.05 mg biomass g^−1^ H_2_O in lower water potentials, a 70% decrease. At LET, these values were 2.44 and 0.81 mg biomass g^−1^ H_2_O, respectively, a 67% decrease.

## 4. Discussion

In this study, the primary objective was to determine the drought threshold which impacts rice growth. We validated the threshold value in two independent greenhouse experiments conducted during middle summer and fall, when high (HET) and low (LET) evapotranspiration conditions prevailed. Rice is mostly grown in flooded areas, although there is some successful water management withdrawing the continuous flooding [14]. The water saving technology has been proposed through alternate wetting and drying cycles [13,14,15,16,34], saturated soil without water ponding [10,11,12], or aerobic rice [4,35]. Regardless, the irrigation approach is primordial and does not negatively impact the crop evapotranspiration, and it is essential to know the critical water potential in the soil that limits yield [21]. For this purpose, our study established a water potential threshold which can be used in further studies to investigate rice tolerance to water deficit, and also in new water-saving cultivation systems.

Evapotranspiration and leaf area were the parameters affected in the least negative potentials (Figure 1). Evapotranspiration began decreasing when the water potential was −0.046 MPa on HET and −0.047 MPa on LET. Leaf area decreased when the solution’s water potential was less than −0.046 MPa on HET and −0.053 MPa on LET. The aboveground biomass (Figure 2) showed a decrease when the water potential was less than −0.056 in HET, and −0.057 in LET plants. Root biomass also decreased in HET plants when water potential was lower than −0.063 MPa.

Our data showed that rice is very sensitive to drought independently of the evapotranspiration conditions (LET and HET). All evaluated parameters showed that HET plants performed better than LET plants in the nutrient solution with no PEG addition (−0.04 MPa). Besides, a lower relative humidity and higher evapotranspiration, the temperature was higher during HET period (see Table 1). Additionally, although we have not measured irradiance, light intensity was surely higher at HET period than LET period, since the first occurred during the Brazilian summer (December to March) and the latter during the autumn (March to June). Thus, the better performance of HET plants might be expected, but curiously, the water potential in which all evaluated parameters dropped were very similar for both HET and LET evapotranspiration periods.

Drought induces several morphological changes in rice, such as a decrease in mass accumulation in roots and shoots, resulting in a decrease in plant productivity. However, these changes may vary according to the timing the stress is applied [36]. Drought stress imposed in the terminal stage of rice cycle is the most detrimental to grain yield, while plants may recover when the stress is applied in the vegetative stage [37]. This study was carried out as a refinement of the research of Reis et al. [4], who studied different irrigation systems in lowland rice in Brazil. In the aerobic systems, these authors observed that when the soil water potential dropped between −0.05 to −0.06 MPa there was a decrease in productivity. These values were attained during the vegetative (V4–V5) and reproductive (R0–R4) stages. When the water potential was around −0.02 to −0.03 MPa, productivity was maintained. The lowest water potential observed by Reis et al. [4] are close to the values we observed here (−0.046 to −0.056 MPa) for rice plants grown in nutritive solutions containing PEG. Considering the results of Reis et al. [4] obtained in the field with lowland rice, that grain production can be seen as a partial function of WUE and ET [38] and that both parameters decreased in our experiments (WUE, Table 2 and ET, Figure 1) in both periods (HET and LET), we suggest that this water potential range is critical for growth and productivity of lowland rice. The definition of a critical value may help to improve water management in rice cultivation and to keep productivity. We are aware, however, that the values we obtained here may not be definitive depending on the variety and developmental stage.

## Figures and Tables

**Table 1:** Climate parameters during high evapotranspiration (HET) and low evapotranspiration (LET) growing cycles. T_max_ = average of maximum temperature; T_min_ = average of minimum temperature; T_av_ = average temperature; RH = relative humidity; ET_0_ = potential evapotranspiration calculated by the method of Penman–Monteith (FAO 56 PM).

Period	T_max_	T_min_	T_av_	RH	ET_0_
	------------- (°C) ------------			(%)	(mm)
HET	32	20	25	76	622
LET	26	15	20	82	289

**Table 2:** Water use efficiency (WUE) in rice plants grown at different solution water potentials and at HET and LET.

Solution Water Potential	HET	LET
(MPa)	(mg biomass g^−1^ H_2_O)	
−0.04	3.47 a	2.44 a
−0.07	0.83 b	0.79 b
−0.10	1.06 b	0.73 b
−0.13	1.07 b	0.96 b
−0.16	1.41 b	0.87 b
−0.19	0.89 b	0.71 b

Values in the same column followed by the same letter are not significantly different (*p*-value < 0.05).
